# Research Derived From Medicare’s Coverage With Evidence Development Program

**DOI:** 10.1001/jamanetworkopen.2025.5077

**Published:** 2025-04-15

**Authors:** Guneet S. Janda, Osman Moneer, Maryam Mooghali, Reshma Ramachandran, Joshua D. Wallach, Sanket S. Dhruva, Joseph S. Ross

**Affiliations:** 1 Department of Medicine, Mayo Clinic School of Medicine, Rochester, Minnesota; 2 Department of Medicine, Stanford University School of Medicine, Palo Alto, California; 3 Department of Medicine, Yale School of Medicine, New Haven, Connecticut; 4 Section of General Medicine and Yale Collaboration for Regulatory Rigor, Integrity and Transparency, Yale School of Medicine, New Haven, Connecticut; 5 Department of Epidemiology, Rollins School of Public Health, Emory University, Atlanta, Georgia; 6 Department of Medicine, University of California, San Francisco, School of Medicine, San Francisco; 7 San Francisco Veterans Affairs Health Care System, San Francisco, California; 8 Department of Health Policy and Management, Yale School of Public Health, New Haven, Connecticut; 9 Center for Outcomes Research and Evaluation, Yale-New Haven Hospital, New Haven, Connecticut

## Abstract

This cross-sectional study assesses published research articles about medical items and services approved by the Coverage With Evidence Development program to characterize their scientific impact.

## Introduction

Medicare’s Coverage with Evidence Development (CED) program requires beneficiary participation in clinical studies as a condition of coverage for an item or service.^1^ Since CED’s initiation in 2005, concerns have been raised about coverage requirements and associated costs.^2,3^ However, CED offers a trade-off: beneficiary access to novel technologies and manufacturer access to the Medicare market in exchange for research addressing product benefits and harms.^4^ CED may also have positive externalities, generating evidence to inform care decisions beyond Medicare policy, including for the Food and Drug Administration (FDA).^4,5^ In this study, our objective was to identify how many published research articles were derived from CED-approved studies and characterize their scientific impact.

## Methods

In accordance with the Common Rule, this study was exempt from ethics review and informed consent because it was not human participant research. We followed the STROBE reporting guideline.

We used the Centers for Medicare & Medicaid Services (CMS) website to identify all items and services with CED program requirements as of July 1, 2023.^6^ For each, we determined therapeutic area and all CED-approved studies, including National Clinical Trial numbers. For each study, we extracted study type (registry vs nonregistry) and completion status from ClinicalTrials.gov. We used multiple search strategies to identify all published English-language original research and systematic reviews that reported results from these CED-approved studies as of July 1, 2023, excluding commentaries and editorials; ClinicalTrials.gov, PubMed, and Google Scholar were searched. For each research publication, we used Scopus to determine number of times cited and proportion cited by clinical practice guidelines as of February 2024. We reviewed FDA product labels to ascertain whether research from CED-approved studies was cited as of March 2024.

Additional study details are provided in eMethods in Supplement 1. Descriptive statistics were used to summarize key characteristics, analyzed in Excel, version 16.63.1 (Microsoft Corp).

## Results

As part of the CED program, CMS approved 116 unique studies for 26 items and services, including 19 medical devices (73.1%), 5 blood- or tissue-derived products (19.2%), and 2 drugs (7.7%). As of July 2023, 32 CED-approved studies (27.6%) were complete and 26 (22.4%) were active but no longer recruiting; 68 (58.6%) were in cardiology, while 86 (74.1%) were clinical trials, 23 (19.8%) registries, 4 (3.4%) claims-based studies, and 3 (2.6%) expanded access studies (Table 1).

**Table 1.** Medicare-Approved Studies for Items and Services With Coverage With Evidence Development Requirements and Associated Research Publications, Overall and Stratified by Therapeutic Area, Study Type, and Study Completion Status

Characteristic	Studies, No. (%)		Research publications, No. (%)	Research publications per study, median (IQR)
	CMS-approved studies	CMS-approved studies with at least 1 research publication		
Overall	116 (100)	53 (45.7)	556 (100)	0 (0-3)
Therapeutic area				
Cardiology	68 (58.6)	30 (44.1)	454 (81.7)	0 (0-3.8)
Hematology	20 (17.2)	5 (25.0)	14 (2.5)	0 (0-0.5)
Musculoskeletal	0	0	0	0
Neurology	10 (8.6)	5 (50.0)	20 (3.6)	0 (0-3)
Oncology	10 (8.6)	8 (80.0)	58 (10.4)	1 (0.75-12)
Otolaryngology	5 (4.3)	2 (40.0)	2 (0.4)	0 (0-1)
Pulmonology	2 (1.7)	2 (100)	7 (1.3)	3.5 (2-5)
Psychiatry	1 (0.9)	1 (100)	1 (0.2)	1 (1-1)
Study type				
Clinical trial	86 (74.1)	43 (50.0)	267 (48.2)	0 (0-2)
Registry	23 (19.8)	8 (34.8)	281 (50.4)	0 (0-4)
Claims-based study	4 (3.4)	1 (25.0)	4 (0.7)	0 (0-3)
Expanded access	3 (2.6)	1 (33.3)	4 (0.7)	0 (0-4)
Study completion status^a^				
Complete	32 (27.6)	21 (65.6)	122 (21.9)	1 (0-4.8)
Active, not recruiting	26 (22.4)	17 (65.4)	143 (25.7)	2 (0-5.8)
Recruiting	26 (22.4)	9 (34.6)	262 (47.1)	0 (0-2.5)
Not yet recruiting	1 (0.9)	0	0	0
Suspended	1 (0.9)	0	0	0
Terminated	14 (12.1)	4 (28.6)	9 (1.6)	0 (0-1)
Unknown status	16 (13.8)	2 (12.5)	20 (3.6)	0 (0-0)

Abbreviation: CMS, Centers for Medicare and Medicaid Services.

^a^ As of July 1, 2023.

Results from 57 CED-approved studies (49.1%) had been reported as of July 2023, including 53 (45.7%) with 1 or more research publications and 4 (3.4%) reporting results only on ClinicalTrials.gov, with higher rates among CED-approved studies that were completed (21 [65.6%]) and active but no longer recruiting (17 [65.4%]) (Table 1). In total, there were 556 unique research publications, including 267 (48.2%) from CED-approved clinical trials and 281 (50.4%) from CED-approved registries (Table 2). Clinical trial publications were cited a median (IQR) of 22 (6-61) times, and 76 (28.5%) were cited by clinical practice guidelines. Registry publications were cited a median (IQR) of 25 (10-65.5) times, with 123 (43.8%) cited by clinical practice guidelines and 1 (0.4%) by the FDA.

**Table 2.** Characteristics of Research Publications Associated With Items and Services With Coverage With Evidence Development Requirements, Stratified by Medicare-Approved Study Type

Characteristic	Research publications, No. (%)		
	Derived from clinical trial studies (n = 267)	Derived from registry studies (n = 281)	Derived from claims-based or expanded access studies (n = 8)
Therapeutic area			
Cardiology	183 (68.5)	263 (93.6)	8 (100)
Hematology	12 (4.5)	2 (0.7)	0
Musculoskeletal	0	0	0
Neurology	16 (6.0)	4 (1.4)	0
Oncology	46 (17.2)	12 (4.3)	0
Otolaryngology	2 (0.7)	0	0
Pulmonology	7 (2.6)	0	0
Psychiatry	1 (0.4)	0	0
Study completion status^a^			
Complete	118 (44.2)	4 (1.4)	0
Active, not recruiting	123 (46.1)	12 (4.3)	8 (100)
Recruiting	9 (3.4)	253 (90.0)	0
Not yet recruiting	0	0	0
Suspended	0	0	0
Terminated	9 (3.4)	0	0
Unknown status	8 (3.0)	12 (4.3)	0
Cited by FDA	0	1 (0.4)	0
Cited by clinical practice guideline in Scopus	76 (28.5)	117 (41.6)	1 (12.5)
Citation count, median (IQR)	22 (6-61)	25 (10-65.5)	23 (6-45)
Time since publication to July 1, 2023, median (IQR), mo	36 (18-67)	64 (39-103)	21 (15-54)

Abbreviation: FDA, Food and Drug Administration.

^a^ As of July 1, 2023.

## Discussion

In this cross-sectional analysis of all 26 items and services with CED program requirements, approximately half of 116 CED-approved studies had reported results as of July 2023, totaling 556 unique publications, even though fewer than one-third had been completed. These publications were widely cited, including by clinical practice guidelines. We cannot conclude with certainty that these studies would have been conducted without CED, and CED-approved study completion and results reporting are suboptimal. Nevertheless, the findings suggest that CED contributes not only to CMS decision-making but also the medical research enterprise.

Study limitations include possible completion of some CED-approved studies and publication of additional articles since our July 2023 search. Moreover, despite using multiple publication search strategies, we may have missed some studies, and some might have been used by clinical practice guidelines or the FDA but not cited, rendering our estimates conservative. Publication and citation numbers are imperfect measures of scientific impact.

## Notes

Supplement 1.
eMethods

eReferences

Supplement 2.
Data Sharing Statement

## References

[1] Centers for Medicare & Medicaid Services. Guidance for the public, industry, and CMS staff: Coverage with Evidence Development. Accessed February 8, 2025. https://www.cms.gov/medicare-coverage-database/view/medicare-coverage-document.aspx?MCDId=27

[2] Zeitler EP, Gilstrap LG, Coylewright M, Slotwiner DJ, Colla CH, Al-Khatib SM. Coverage with Evidence Development: where are we now? Am J Manag Care. 2022;28(8):382-389. doi:10.37765/ajmc.2022.88870 35981123

[3] Lakdawalla D, Tunis S, Neumann P, Whicher D, Zeitler E, Liden B. A roadmap for improving Medicare’s application of Coverage with Evidence Development. Value Health. 2024;27(9):1191-1195. doi:10.1016/j.jval.2024.05.008 38795958

[4] Dhruva SS, Ramachandran R, Ross JS. Medicare’s national coverage determination for aducanumab - a one-off or a pragmatic path forward? N Engl J Med. 2022;387(17):1539-1541. doi:10.1056/NEJMp2210198 36301560

[5] Mooghali M, Moneer O, Janda G, Dhruva SS, Ross JS, Ramachandran R. Assessing Medicare’s Coverage with Evidence Development program. Health Aff (Millwood). 2025;44(1):32-39. doi:10.1377/hlthaff.2024.00814 39761464

[6] Centers for Medicare & Medicaid Services. Coverage with Evidence Development. Accessed February 8, 2025. https://www.cms.gov/medicare/coverage/evidence

