# Supplementary material for: Research Derived From Medicare’s Coverage With Evidence Development Program
Source: JAMA Netw Open. 2025 Apr 15;8(4):e255077. doi: 10.1001/jamanetworkopen.2025.5077 (PMC12000966; doi:10.1001/jamanetworkopen.2025.5077)
Supplement: Supplement 1. — eMethods eReferences [file jamanetwopen-e255077-s001.pdf]

Supplemental Online Content

Janda GS, Moneer O, Mooghali M, et al. Research derived from Medicare's Coverage with Evidence Development program. *JAMA Netw Open*. 2025;8(4):e255077. doi:10.1001/jamanetworkopen.2025.5077

eMethods

eReferences

This supplemental material has been provided by the authors to give readers additional information about their work.

© 2025 Janda GS et al. *JAMA Network Open*

eMethods

Identification of CED Designations and Approved Studies

We used the CMS website to identify all items and services with a CED requirement as part of an NCD as of July 1, 2023.[1] For each CED designation, we identified all CED-approved studies listed on the CMS website and confirmed the accuracy of the final list through communication with CMS. For each CED designation, we characterized the therapeutic area and extracted the benefit category, purpose of item or service (therapeutic or diagnostic), and status of CED requirement as of July 1, 2023.

For each CED-approved study, we extracted the CMS approval year and National Clinical Trial (NCT) number from the CMS website. We also extracted the study sponsor, study type (randomized controlled trial [RCT], registry, claims-based study, or expanded access study), and completion status (complete, active - not recruiting, recruiting, not yet recruiting, suspended, terminated, unknown) from ClinicalTrials.gov.

Identification of Publications

We used multiple search strategies to identify all English-language original research and systematic review articles published in peer-reviewed journals that reported on analyses using data generated from approved clinical studies, excluding perspectives, commentaries, and editorials.

First, we examined all publications listed as part of the ClinicalTrials.gov registration for a given CED-approved study, including those provided by the responsible party and those automatically hyperlinked via PubMed. We then manually searched PubMed and Google Scholar using the NCT numbers. We also attempted to find any centralized listings or databases of

© 2025 Janda GS et al. *JAMA Network Open*

published articles results from the studies, which are typically maintained by trial teams or sponsors. All publication searches were time-bound between the CMS approval date for the specific clinical study through an online publication date of July 1, 2023.

One investigator (G.S.J.) screened all articles at the title and abstract level, then evaluated potentially eligible publications at the full-text level. Two other investigators (O.M. and M.M.) each validated a random non-overlapping 5% of the sample. Any uncertainties were resolved by discussion to achieve consensus, engaging additional team members as needed.

Data Abstraction from Publications

For all research publications resulting from CED-approved studies, we extracted the corresponding author affiliation (academic, government, industry, other) and primary end point(s) or objective. We then determined the type of analysis for both registry and non-registry publications, outlined as follows. For publications identified from CED-approved non-registry studies (predominantly clinical trials), we determined whether they reported the primary analysis of a study (defined as an analysis that examined the primary end point(s) in the entire patient cohort as registered on ClinicalTrials.gov), secondary analyses of a study (defined as any analysis that examined a secondary end point or that examined the primary end point(s) in a subgroup of the overall patient cohort), systematic reviews that included the study (with or without meta-analysis), or study design reports. Publications identified from CED-approved registries were assessed separately from non-registry studies because they often do not have clearly defined primary end points or study durations. Publications identified from CED-approved registry studies were categorized as comparative analyses of patient cohorts, descriptive (i.e., non-comparative) clinical analyses, assessments of a model or mining of

© 2025 Janda GS et al. *JAMA Network Open*

predictive factors, systematic reviews that included the study (with or without meta-analyses), utilization or cost effectiveness analyses, or other.

Citation of Publications

For each research publication, we then assessed the citation count (i.e., number of times cited in other peer-reviewed publications as determined through Scopus as of February 2024) and number of times cited by clinical practice guidelines published by professional societies in peer-reviewed journals as determined through Scopus. Finally, using the FDALabel search database, we conducted full-text searches of drug, biologic, and device labels to determine if any research publications resulting from CED-approved studies were cited in any product labels as of March 2024. We also searched all FDA product safety communications and letters to health care providers and determined if any publications from CED-approved studies were cited.

Statistical analysis

Descriptive statistics were used to summarize key characteristics across CED-approved studies, including number and types of publications by CED-approved study completion status (e.g., completed vs ongoing), product type (i.e., therapeutic vs diagnostic), therapeutic area, and study type (i.e., clinical trials vs observational studies). Data were collected and analyzed in Microsoft Excel (Version 16.63.1).

© 2025 Janda GS et al. *JAMA Network Open*

eReferences

1. Centers for Medicare & Medicaid Services (CMS). Coverage with Evidence Development. Last accessed on February 8, 2025 at https://www.cms.gov/medicare/coverage/evidence.
2. Fang H, Harris S, Liu Z, et al. FDALabel for drug repurposing studies and beyond. *Nat Biotechnol*. 2020/12/01 2020;38(12):1378-1379. doi:10.1038/s41587-020-00751-0
3. U.S. Food and Drug Administration. FDALabel: Full-text Search of Drug Labeling. Last accessed on February 8, 2025 at https://www.fda.gov/science-research/bioinformatics-tools/fdalabel-full-text-search-drug-product-labeling.

© 2025 Janda GS et al. *JAMA Network Open*
