# Supplementary material for: Research Derived From Medicare’s Coverage With Evidence Development Program
Source: JAMA Netw Open. 2025 Apr 15;8(4):e255077. doi: 10.1001/jamanetworkopen.2025.5077 (PMC12000966; doi:10.1001/jamanetworkopen.2025.5077)
Supplement: Supplement 2. — Data Sharing Statement [file jamanetwopen-e255077-s002.pdf]

Data Sharing Statement

Janda. Research Derived from Medicare's Coverage With Evidence Development Program. *JAMA Netw Open*. Published April 15, 2025. doi:10.1001/jamanetworkopen.2025.5077

Data
Data available: Yes
Data types: Data (not involving human participants)
How to access data: Data will be made available via the corresponding author: joseph.ross@yale.edu.
When available: With publication

Supporting Documents
Document types: None

Additional Information
Who can access the data: Anyone requesting the data
Types of analyses: For any purpose
Mechanisms of data availability: Without investigator support
